# Prognostic nutritional index (PNI) is an independent predictor for functional outcome after hip fracture in the elderly: a prospective cohort study

**DOI:** 10.1007/s11657-024-01469-1

**Published:** 2024-11-05

**Authors:** Yimin Chen, Mingjian Bei, Gang Liu, Jing Zhang, Yufeng Ge, Zhelun Tan, Weidong Peng, Feng Gao, Chao Tu, Maoyi Tian, Minghui Yang, Xinbao Wu

**Affiliations:** 1 Department of Orthopedics and Traumatology, Peking University Fourth School of Clinical Medicine, Beijing, China; 2 Beijing Jishuitan Hospital, Capital Medical University, Beijing, China; 3 National Center for Orthopedics, Beijing, China; 4 School of Public Health, Harbin Medical University, Harbin, Heilongjiang, China; 5 The George Institute for Global Health, University of New South Wales, Sydney, New South Wales Australia; 6 Department of Orthopedics and Traumatology, Peking University Fourth School of Clinical Medicine, Beijing Jishuitan Hospital, National Center for Orthopaedics, #31 Xinjiekou East Road, Beijing, 100035 China

**Keywords:** Hip fracture, Osteoporosis, Nutrition, Prognostic nutritional index, Mobility, Health-related quality of life

## Abstract

***Summary*:**

The prognostic nutritional index (PNI) is a useful tool for assessing nutritional status using serum albumin and lymphocyte count. This study indicates that a higher preoperative PNI correlates with improved mobility and health-related quality of life during the initial postoperative period in elderly patients with hip fractures.

**Purpose:**

To investigate the prognostic value of the prognostic nutritional index (PNI) in predicting mobility and health-related quality of life (HRQoL) in elderly hip fracture patients after surgery.

**Methods:**

We prospectively involved patients aged 65 and above, who could walk freely before injury and underwent surgery between 2018 and 2019. Admission PNI was calculated as serum albumin (g/L) + 5 × total lymphocyte count (× 10^9^/L). Patients were classified into two groups based on PNI median value. All patients were followed up by telephone for four times (30-day, 120-day, 1-year, and 3-year after surgery). The Fracture Mobility Score (FMS) and EuroQol 5-Dimension 5-Level (EQ-5D 5L) were used to evaluate mobility and HRQoL, respectively.

**Results:**

Of 705 eligible patients, 487 completed all assessments. Patients in the higher PNI group had a significantly increased possibility of achieving unrestricted mobility at the 120-day follow-up (OR 1.69, 95% CI 1.10–2.61, P.adj = 0.017), while no significant differences were observed at other follow-ups. Additionally, patients in the higher PNI group had a significantly higher EQ-5D utility value at the 30-day follow-up (P.adj = 0.015). A linear regression model with adjusting for all confounders showed that admission PNI value was positively associated with EQ-5D utility values at 30-day, 120-day, and 1-year follow-up assessments (P.adj = 0.011, P.adj = 0.001, and P.adj = 0.030, respectively). However, this correlation was not observed at the 3-year time point (P.adj = 0.079).

**Conclusion:**

The PNI is a valuable predictor of functional outcomes in elderly patients with hip fractures following surgery.

**Supplementary Information:**

The online version contains supplementary material available at 10.1007/s11657-024-01469-1.

## Introduction

Hip fractures in the elderly are associated with decreased mobility and independence [1, 2]. Malnutrition is common in elder patients with hip fracture, leading to poor outcomes, higher complication rates, reduced mobility recovery, and increased mortality [3, 4]. It is important to identify elderly hip fracture patients at the risk of malnutrition in hospital setting as early as possible for optimal nutritional care [5].

Up to now, various nutritional screening tools (NSTs) have been developed to evaluate patients’ nutritional status, including Subjective Global Assessment (SGA) [6], Malnutrition Universal Screening Tool (MUST) [7], and Mini Nutritional Assessment (MNA) [8]. However, these NSTs are usually complex and some of them are subjective assessments which require skill and experience. An objective and easy tool for estimating nutritional status in geriatric hip fracture patients is still needed.

The prognostic nutritional index (PNI) is a simple tool for evaluating perioperative nutritional status calculated by preoperative serum albumin (Alb) and total lymphocyte count (TLC). A low PNI level has been identified as a significant predictor of poor outcomes in various diseases, including gastrointestinal cancer (PNI < 40) [9], colorectal cancer (PNI < 45.5) [10], lung cancer (PNI < 45.5) [11], chronic obstructive pulmonary disease (COPD) (PNI < 48.84) [12], and diabetic nephropathy[13]. In addition, studies had reported that a lower preoperative PNI was associated with significantly higher mortality in hip fracture patients [14]. However, few studies had investigated the prognostic value of PNI for postoperative mobility and health-related quality of life (HRQoL) in Chinese geriatric hip fracture patients. In this study, we aimed to assess the potential association between the PNI and functional outcomes after surgery for hip fracture in the elderly.

## Methods

### Study design

The current research was carried out at a tertiary hospital in Beijing, China, where a collaborative orthogeriatric hip fracture care pathway was introduced. Approval for the study was obtained from the Institutional Review Board at Peking University Health Science Center (IRB00001052-17021) and the Biomedical Ethics Committee at Beijing Jishuitan Hospital (201807-11). All participants provided written consent before data collection. The post hoc analysis used baseline data from a previous observational study in China that examined the impact of this co-management model on elderly patients with hip fractures (Clinical Trials.gov Identifier: NCT03184896) [15].

### Study population, recruitment, and follow-up

The current study included patients aged 65 years and older with X-ray confirmed hip fracture within 3 weeks after injury between November 26, 2018, and November 30, 2019. A total of 1057 patients who had undergone surgery for femoral neck fractures (FNFs) or intertrochanteric fractures (ITFs) were screened. The clinical approach used has been previously described [15]. During the screening process, the patients who met the following exclusion criteria would be ruled out: (1) those who were unable to walk freely without an assistive device before injury; (2) those lacked completed baseline data including laboratory test at admission; (3) those with pathological fractures; (4) those with terminal malignancies.

**Fig. 1:**
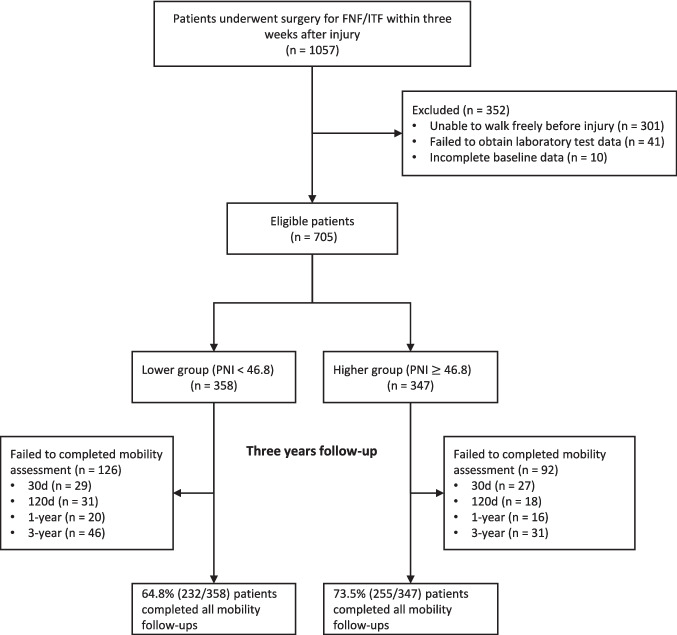
Flowchart of the study. Patients who could not walk freely without aid before injury were excluded. Eligible patients were classified into two groups based on the median of admission PNI value (46.8). Abbreviations: FNF, femoral neck fracture; ITF, intertrochanteric fracture; PNI, prognostic nutritional index

Figure 1 presents the study’s flowchart. After excluding 352 patients, 705 were enrolled. PNI was calculated using initial blood test results with the formula: serum Alb (g/L) + 5 × TLC (× 10^9^/L) [9]. Among these patients, PNI values ranged from 32.8 to 60.0, with a mean ± standard deviation (SD) of 46.6 ± 4.2. Then, patients were categorized into two groups based on median of the PNI values: the lower (< 46.8) and the higher (≥ 46.8). All participants were followed up by telephone from orthopedists for four times (30-day, 120-day, 1-year, and 3-year after surgery).

### Data collection

The present study prospectively collected demographic and perioperative data. Socio-demographic variables involved age, sex, body mass index (BMI), smoking and drinking habits, and education level. Comorbidities at baseline including hypertension, diabetes, anemia, apoplexy, coronary artery disease (CAD), depression, history of malignant disease, acute pneumonia, Parkinson’s disease, and cognitive and visual impairments were also documented. Cognitive ability was evaluated using the Mini-Mental State Examination-China (MMSE), and participants with an MMSE score of 23 or lower were considered to have cognitive impairment [16]. Educational level was classified into four levels, ranging from illiterate to university or higher. The overall medical condition was represented by the Charlson Comorbidity Index (CCI). The number of falls of patients in the past year was used to reflect the tendency to be injured. Cardiac function was indicated by the left ventricular ejection fraction (LVEF) obtained from preoperative echocardiography conducted in the emergency department (ED). Perioperative variables encompassed fracture type, American Society of Anesthesiologists (ASA) scores, type of anesthesia and operation, rehabilitation, and length of stay (LOS). The results of blood routine and biochemical test at admission were also collected. Femoral neck fractures were treated with osteosynthesis or arthroplasty, while intertrochanteric fractures were treated with intramedullary nailing, dynamic hip screw (DHS), or locking plate. Operations were categorized into two groups: (1) internal fixation (cannulated screw fixation, intramedullary nailing, DHS, and locking plate) and (2) arthroplasty (hemiarthroplasty and total hip arthroplasty).

Follow-up information included mobility and HRQoL. Mobility was assessed by the Fracture Mobility Score (FMS) from the UK’s “Blue Book” which was adopted by the National Hip Fracture Database (NHFD). The FMS classified patients’ mobility into five levels: freely mobile without aids; mobile outdoors with one aid; mobile outdoors with two aids or frame; some indoor mobility but never goes outside without help; no functional mobility (using lower limbs) [17]. In the current study, we especially focus on the proportion of patients who regained the ability of walking freely at each follow-up time point. The Health-Related Quality of Life (HRQoL) was assessed using the EuroQol 5-Dimension 5-Level (EQ-5D 5L) instrument, which features a five-level response scale (ranging from no issues to severe issues) across five domains pertaining to daily functioning: mobility, self-care, usual activities, pain and discomfort, and anxiety and depression [18]. The responses were then converted into an overall score using a published utility model for the Chinese population [19].

### Study outcomes

The study only included patients who completed all four follow-up assessments for the final analysis. The primary outcome was postoperative mobility, comparing the proportion of patients able to walk without aids between two groups at various follow-up time points. The secondary outcome was EQ-5D utility values, comparing averages between groups at each follow-up interval. Additionally, correlation between admission PNI value and EQ-5D utility value was analyzed in all patients.

### Statistical analysis

For baseline data, parametric data are described using means and SDs or medians and interquartile ranges (IQRs). Categorical data are shown as frequencies and percentages. The chi-squared test was used for categorical variables, while Student’s *t*-test or Mann–Whitney *U*-test was used for continuous variables based on parametric or non-parametric data. Variables with *P* < 0.05 from univariable analysis were considered confounders. Logistic regression models were used to compare the mobility between groups at different follow-up times with or without adjusting for confounders. In addition, we employed the generalized estimating equations (GEE) method to assess the difference in EQ-5D utility between two groups at various follow-up time intervals. A multivariate linear regression model with adjusting for all covariates was also used to assess the association between the admission PNI value and the EQ-5D utility value at each follow-up.

The analyses were conducted using the statistical software packages R 4.1.1 (http://www.R-project.org, The R Foundation). A two-tailed test was utilized, with statistical significance defined as *P* < 0.05.

## Results

### Population and baseline characteristics

There were 705 eligible patients included in our study. Table 1 summarizes the baseline characteristics of them. The mean age was (78.2 ± 7.5) years old, and 70.8% patients were female. Among the patients, 54.3% had FNF, and 76.3% received surgery within 48 h after admission. Patients in the lower PNI group (80.1 ± 7.5) were older than those in the higher PNI group (76.2 ± 7.1). Patients in the lower PNI group had a lower BMI value [(22.2 ± 3.9) vs (23.4 ± 3.5), *P* < 0.001]. Patients in the lower PNI group had a lower proportion of diabetes (21.5% vs 34.9%, *P* < 0.001), hypertension (56.1% vs 64.6%, *P* = 0.023), but a higher proportion of anemia (55.0% vs 25.4%, *P* < 0.001) and cognitive impairment (11.5% vs 4.6%, *P* < 0.001). The proportion of patients with a smoking history was higher in the lower PNI group (20.1% vs 12.4%, *P* = 0.006). Patients in the lower PNI group had a higher proportion of ITF (53.4% vs 37.8%, *P* < 0.001). Additionally, the LOS was longer in the lower PNI group compared to the higher PNI group (*P* = 0.019). In our final analysis, 487 patients (46.1%, 487/1,057) completed all four follow-ups during 3 years, with 232 patients in the lower PNI group and 255 in the higher PNI group (Fig. 1).

**Table 1:** Baseline characteristics

Variables	Total (*n* = 705)	Lower (*n* = 358)	Higher (*n* = 347)	*P*-value
Age, years, Mean ± SD	78.2 ± 7.5	80.1 ± 7.5	76.2 ± 7.1	< 0.001
Sex, *n* (%)				< 0.001
Female	499 (70.8)	229 (64)	270 (77.8)	
Male	206 (29.2)	129 (36)	77 (22.2)	
BMI, kg/m^2^, Mean ± SD	22.8 ± 3.7	22.2 ± 3.9	23.4 ± 3.5	< 0.001
Comorbidity, *n* (%)				
Diabetes	198 (28.1)	77 (21.5)	121 (34.9)	< 0.001
Hypertension	425 (60.3)	201 (56.1)	224 (64.6)	0.023
Anemia	285 (40.4)	197 (55.0)	88 (25.4)	< 0.001
Apoplexy	162 (23.0)	84 (23.5)	78 (22.5)	0.756
CAD	196 (27.8)	101 (28.2)	95 (27.4)	0.805
Depression	11 (1.6)	7 (2)	4 (1.2)	0.390
Acute pneumonia	21 (3.0)	11 (3.1)	10 (2.9)	0.882
Parkinson’s disease	20 (2.8)	14 (3.9)	6 (1.7)	0.081
Visual impairment	272 (39.0)	135 (38)	137 (39.9)	0.604
Cognitive impairment	57 (8.1)	41 (11.5)	16 (4.6)	< 0.001
CCI, *n* (%)				0.289
0	246 (34.9)	125 (34.9)	121 (34.9)	
1	250 (35.5)	128 (35.8)	122 (35.2)	
2	127 (18.0)	57 (15.9)	70 (20.2)	
≥ 3	82 (11.6)	48 (13.4)	34 (9.8)	
Ever or current smoker, *n* (%)	115 (16.3)	72 (20.1)	43 (12.4)	0.006
Current drinker, *n* (%)	35 (5.0)	19 (5.3)	16 (4.6)	0.670
Live alone, *n* (%)	81 (11.6)	40 (11.2)	41 (11.9)	0.778
MMSE, Mean ± SD	20.9 ± 5.2	20.1 ± 5.8	21.8 ± 4.2	< 0.001
Education level, *n* (%)				0.044
Illiterate	117 (16.6)	68 (19.0)	49 (14.1)	
Primary school or lower	162 (22.9)	92 (25.7)	70 (20.2)	
High school	212 (30.1)	97 (27.1)	115 (33.1)	
University or higher	214 (30.4)	101 (28.2)	113 (32.6)	
Falling times in the last year, n (%)				0.419
0	304 (43.1)	146 (40.8)	158 (45.5)	
1	326 (46.2)	171 (47.8)	155 (44.7)	
≥ 2	75 (10.7)	41 (11.4)	34 (9.8)	
Non-ground level fall, *n* (%)	95 (13.7)	46 (13)	49 (14.4)	0.608
TTS ≤ 48 h, *n* (%)	538 (76.3)	269 (75.1)	269 (77.5)	0.457
Fracture type, *n* (%)				< 0.001
FNF	383 (54.3)	167 (46.6)	216 (62.2)	
ITF	322 (45.7)	191 (53.4)	131 (37.8)	
Fracture side, *n* (%)				0.957
Left	347 (49.2)	178 (49.7)	169 (48.7)	
Right	350 (49.6)	176 (49.2)	174 (50.1)	
Bilateral	8 (1.1)	4 (1.1)	4 (1.2)	
LVEF, Mean ± SD	65.5 ± 4.9	65.2 ± 4.8	65.8 ± 4.9	0.161
ASA, *n* (%)				0.251
I	105 (14.9)	53 (14.8)	52 (15)	
II	358 (50.8)	172 (48)	186 (53.6)	
≥ III	242 (34.3)	133 (37.2)	109 (31.4)	
Anesthesia type, *n* (%)				0.490
Spinal	680 (96.5)	347 (96.9)	333 (96)	
General	25 (3.5)	11 (3.1)	14 (4)	
Operation type, *n* (%)				0.084
Internal fixation	403 (57.2)	216 (60.3)	187 (53.9)	
Arthroplasty	302 (42.8)	142 (39.7)	160 (46.1)	
LOS, days, Median (IQR)	4.9 (4.0, 6.1)	5.0 (4.0, 6.3)	4.8 (3.9, 5.9)	0.019

*BMI*, body mass index; *CAD*, coronary artery disease; *CCI*, Charlson’s comorbidity index; *MMSE*, mini-mental state examination; *TTS*, time to surgery; *FNF*, femoral neck fracture; *ITF*, intertrochanteric fracture; *LVEF*, left centricular ejection fraction; *ASA*, American society of anesthesiologists; *EQ-5D*, EuroQol 5 Dimensions Questionnaire; *LOS*, length of stay

### Primary outcome

**Fig. 2:**
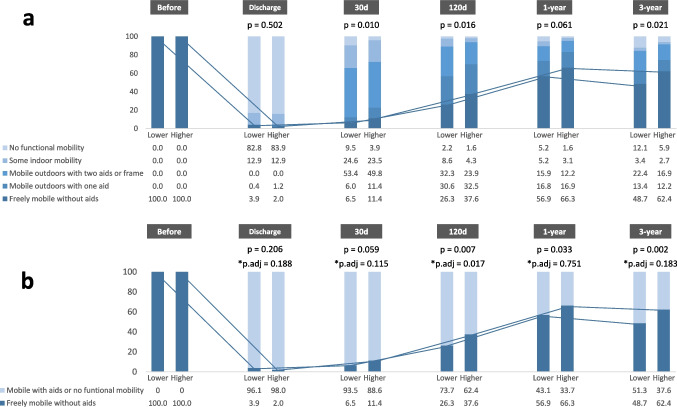
Comparison of postoperative mobility between two groups by assessing the proportion of patients walking without aid at various follow-up time points. Patients were divided into two groups based on the median value of admission PNI (Lower group with PNI < 46.8 and Higher group with PNI ≥ 46.8). **a** The comparison of FMS results between two groups at each time point; **b** the comparison of the proportion of patients who were able to walk independently at each time point. *Calculated by multiple logistic regression models, with adjusting for age, sex, BMI, diabetes, hypertension, cognitive impairment, ever or current smoker, fracture type, anemia, and LOS. The blue lines connecting the columns show how the proportions of patients who could walk freely changed in each group over time. Abbreviations: FMS, fracture mobility score; BMI, body mass index; LOS, length of stay

Figure 2a illustrates the evolution of the distribution of patients with varying levels of mobility as evaluated by FMS throughout the follow-up period. Upon discharge, 3.9% of patients in the lower PNI group and 2.0% in the higher PNI group could walk freely. At the 30-day follow-up, there was no significant difference in the proportion of patients who could walk freely between the two groups. Compared with the lower PNI group, the proportion of patients who were able to walk freely was significantly higher in the higher PNI group at the 120-day, 1-year, and 3-year follow-ups (37.6% vs 26.3%, *P* = 0.007; 66.3% vs 56.9%, *P* = 0.033; and 62.4% vs 48.7%, *P* = 0.002, respectively) (Fig. 2b).

In order to evaluate the independent prognostic value of PNI, penitential covariates were adjusted in logistic regression models. In Model 1, accounting for age, sex, and BMI, individuals in the higher PNI group were significantly more likely to achieve unrestricted mobility at the 120-day follow-up (OR 1.66, 95% CI 1.10–2.51, *P* = 0.016), but no significant differences were found at other time points (30 days, 1 year, and 3 years). In Model 2 with seven additional confounders included in the analysis, patients in the higher PNI group still had a significantly increased probability of achieving unrestricted mobility at the 120-day follow-up (OR 1.69, 95% CI 1.10–2.61; P.adj = 0.017), while no significant differences were observed at other follow-ups (Table 2, Fig. 2b). Notably, age emerged as a key predictor for achieving unrestricted mobility at the later follow-up time points of 1 year and 3 years (OR 0.92, 95% CI 0.90–0.95, P.adj < 0.001; OR 0.87, 95% CI 0.84–0.90, P.adj < 0.001, respectively) (Supplementary Table 1).

**Table 2:** The ORs of PNI for individuals with unrestricted mobility

Group	Able to mobile freely vs unable to mobile freely					
	Unadjusted		Model 1*		Model 2**	
	OR (95% CI)	*P*-value	OR (95% CI)	*P*-value	OR (95% CI)	*P*-value
Discharge						
Lower	Reference		Reference		Reference	
Higher	0.50 (0.15–1.46)	0.210	0.52 (0.15–1.60)	0.270	0.45 (0.13–1.43)	0.188
30-day						
Lower	Reference		Reference		Reference	
Higher	1.86 (0.98–3.64)	0.062	1.93 (0.99–3.92)	0.059	1.77 (0.88–3.69)	0.115
120-day						
Lower	Reference		Reference		Reference	
Higher	1.69 (1.15–2.50)	0.008	1.66 (1.10–2.51)	0.016	1.69 (1.10–2.61)	0.017
1-year						
Lower	Reference		Reference		Reference	
Higher	1.49 (1.03–2.15)	0.034	1.16 (0.78–1.72)	0.470	1.07 (0.70–1.62)	0.751
3-year						
Lower	Reference		Reference		Reference	
Higher	1.74 (1.22–2.51)	0.003	1.31 (0.87–1.97)	0.203	1.35 (0.87–2.09)	0.183

*Model 1, adjusted for age, sex, and BMI

**Model 2, adjusted for Model 1 + diabetes, hypertension, cognitive impairment, ever or current smoker, fracture type, anemia, and LOS

*Abbreviations: OR*, odds ratio; *PNI*, prognostic nutritional index; *BMI*, body mass index; *LOS*, length of stay

### Secondary outcome

**Fig. 3:**
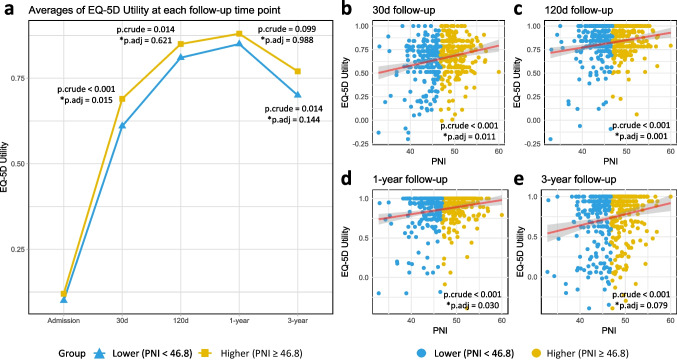
The association between admission PNI value and EQ-5D utility value at various follow-up intervals. **a** Comparison of EQ-5D utility averages between two groups at various follow-up time points (using generalize estimating equations (GEE) method). Patients were divided into two groups based on the median of admission PNI (46.8); **b**–**e** multiple linear regressions of admission PNI value for EQ-5D utility at 30-day, 120-day, 1-year, and 3-year follow-ups. *Adjusted for age, sex, BMI, diabetes, hypertension, cognitive impairment, ever or current smoker, fracture type, anemia, and LOS. Abbreviations: EQ-5D, EuroQol 5 dimensions questionnaire; PNI, prognostic nutritional index; BMI, body mass index; LOS, length of stay

The EQ-5D utility demonstrated a consistent increase during the initial three follow-up periods. Patients in the higher PNI group exhibited notably elevated EQ-5D utility values at the 30-day, 120-day, and 3-year follow-up intervals compared to those in the lower PNI group (*P* < 0.001, *P* = 0.014, and *P* = 0.014, respectively). Nevertheless, upon controlling for ten potential confounding variables, the statistically significant difference persisted solely at the 30-day follow-up (P.adj = 0.015) (Fig. 3a). The results of a multiple linear regression model, which accounted for ten potential covariates, indicated that the admission PNI value exhibited a positive correlation with EQ-5D utility scores at 30-day, 120-day, and 1-year follow-up assessments (P.adj = 0.011, P.adj = 0.001, and P.adj = 0.030, respectively). However, this association was not observed at the 3-year time point (P.adj = 0.079) (Fig. 3b–e).

## Discussion

This study found that among hip fracture patients who were ambulatory before injury, admission PNI is a significant prognostic factor for regaining unrestricted mobility 120 days after surgery. However, age, rather than nutritional status upon admission, emerges as the primary determinant for predicting independent ambulation at 1-year and 3-year postoperative follow-ups. In addition, a positive association was noted between PNI and postoperative EQ-5D utility within the first year following surgery in elderly hip fracture patients.

Malnutrition is very common among the elderly, with around 50% affected [20]. A recent systematic review reported that malnutrition was an independent risk factor for functional dependence and increased mortality in hip fracture patients [21]. A better nutritional care for patients with malnutrition is important to improve their outcomes. Therefore, early and quick identification of patients with malnutrition in the hospital setting remains crucial, especially in the geriatric hip fracture patients [22]. PNI has been reported to correlate significantly with SGA, a widely recognized nutritional screening tool [6, 23, 24]. Although SGA is an inexpensive and quick assessment, it is subjective which requires skill and experiences. In contrast, PNI is fast and objective, which allows surgeons to easily evaluate the immune-nutritional status of elderly hip fracture patients.

Many early studies have reported that PNI has a potential prognostic value in a variety of diseases, including malignancies, COPD, and diabetic nephropathy [9, 12, 13]. In the field of osteoporotic hip fracture, some studies have reported that the PNI is associated with postoperative mortality and delirium in elderly hip fracture patients [14, 25]. However, few studies have investigated the correlation between PNI and functional outcome in geriatric hip fracture patients. Faust et al. found that PNI was an independent predictor of mobility at both three days postoperatively and by discharge (OR 1.14, 95% CI 1.07–1.23, *P* < 0.01; OR 1.18, 95% CI 1.08–1.30, *P* < 0.01; respectively) in geriatric patients with intertrochanteric fractures treated with intramedullary nail osteosynthesis [26]. However, Faust et al. did not perform a long-time follow-up. Studies have reported that only 40 to 60% hip fracture patients could recover their pre-fracture level of mobility [27]. Our research findings suggest that preoperative nutritional status plays a significant role in the initial phase of mobility recovery following surgery. Specifically, patients with a preoperative PNI value greater than 46.8 demonstrated a significantly higher likelihood of achieving unrestricted mobility in the early postoperative period. However, age, instead of preoperative nutritional status, remained the primary predictor for predicting long-term mobility outcomes.

Several studies have established a notable and enduring adverse impact of hip fracture on patients’ HRQoL [28, 29]. However, research within the Chinese healthcare setting is scarce, and there remains a lack of comprehensive understanding regarding the factors that contribute to this lasting effect on HRQoL. This study found that patients with a preoperative PNI value greater than 46.8 exhibited significantly higher EQ-5D utility scores 30 days post-surgery. However, these differences were not sustained at the 120-day, 1-year, and 3-year follow-up assessments. These results suggest that a superior preoperative nutritional status is linked to improved quality of life in the early postoperative period among hip fracture patients. Moreover, our study found a positive correlation between preoperative PNI and postoperative EQ-5D levels within 1 year, but not at the 3-year follow-up, showing the predictive value of PNI for HRQoL in the short term after surgery in geriatric hip fracture patients.

The underlying mechanism of the association between PNI and postoperative mobility and HRQoL remains unclear. PNI indicates a patient’s nutritional and immunological health. Therefore, poor nutrition and weakened immunity could be the main factors leading to adverse outcomes at early stage after surgery [1, 30]. Nonetheless, research suggests that advanced age, rather than nutritional status, plays a more prominent role in determining long-term mobility following surgery.

This study has several limitations. Firstly, it is a single-center cohort study, potentially restricting the applicability of the findings. Secondly, although this study was prospective, participants reported outcomes from memory during each scheduled phone follow-up, potentially causing recall bias. Thirdly, the research was carried out in a hospital situated in a relatively developed area, potentially skewing the patient population towards higher levels of PNI compared to less developed regions. Therefore, conducting multicenter studies could aid in validating the results. Fourthly, despite efforts to adjust for all known confounders in the analysis, there remains the possibility of unmeasured variables influencing the outcomes.

## Conclusion

In conclusion, the prognostic nutritional index (PNI) is a valuable predictor of functional outcomes in elderly patients with hip fractures following surgery.

## Supplementary Information

Below is the link to the electronic supplementary material.

Supplementary file1 (DOCX 25 KB)

## Data Availability

The dataset is managed by Beijing Jishuitan Hospital. The data access request can contact the corresponding author.

## References

[1] Malafarina V, Reginster J-Y, Cabrerizo S, Bruyère O, Kanis JA, Martinez JA et al (2018) Nutritional status and nutritional treatment are related to outcomes and mortality in older adults with hip fracture. Nutrients 10:555. 10.3390/nu10050555 29710860 10.3390/nu10050555 PMC5986435

[2] Li S, Zhang J, Zheng H, Wang X, Liu Z, Sun T (2019) Prognostic Role of serum albumin, total lymphocyte count, and Mini Nutritional Assessment on outcomes after geriatric hip fracture surgery: a meta-analysis and systematic review. J Arthroplasty 34:1287–1296. 10.1016/j.arth.2019.02.003 30852065 10.1016/j.arth.2019.02.003

[3] Bohl DD, Shen MR, Hannon CP, Fillingham YA, Darrith B, Della Valle CJ (2017) Serum albumin predicts survival and postoperative course following surgery for geriatric hip fracture. J Bone Joint Surg Am 99:2110–2118. 10.2106/JBJS.16.01620 29257017 10.2106/JBJS.16.01620

[4] Helminen H, Luukkaala T, Saarnio J, Nuotio M (2017) Comparison of the Mini-Nutritional Assessment short and long form and serum albumin as prognostic indicators of hip fracture outcomes. Injury 48:903–908. 10.1016/j.injury.2017.02.007 28249678 10.1016/j.injury.2017.02.007

[5] Eglseer D, Halfens RJG, Lohrmann C (2017) Is the presence of a validated malnutrition screening tool associated with better nutritional care in hospitalized patients? Nutrition 37:104–111. 10.1016/j.nut.2016.12.016 28359355 10.1016/j.nut.2016.12.016

[6] Detsky AS, McLaughlin JR, Baker JP, Johnston N, Whittaker S, Mendelson RA et al (1987) What is subjective global assessment of nutritional status? JPEN J Parenter Enteral Nutr 11:8–13. 10.1177/014860718701100108 3820522 10.1177/014860718701100108

[7] Stratton RJ, Hackston A, Longmore D, Dixon R, Price S, Stroud M et al (2004) Malnutrition in hospital outpatients and inpatients: prevalence, concurrent validity and ease of use of the “malnutrition universal screening tool” ('MUST’) for adults. Br J Nutr 92:799–808. 10.1079/bjn20041258 15533269 10.1079/bjn20041258

[8] Guigoz Y, Vellas B, Garry PJ (1996) Assessing the nutritional status of the elderly: the Mini Nutritional Assessment as part of the geriatric evaluation. Nutr Rev 54:S59-65. 10.1111/j.1753-4887.1996.tb03793.x 8919685 10.1111/j.1753-4887.1996.tb03793.x

[9] Onodera T, Goseki N, Kosaki G (1984) [Prognostic nutritional index in gastrointestinal surgery of malnourished cancer patients]. Nihon Geka Gakkai Zasshi 85:1001 6438478

[10] Tokunaga R, Sakamoto Y, Nakagawa S, Miyamoto Y, Yoshida N, Oki E et al (2015) Prognostic nutritional index predicts severe complications, recurrence, and poor prognosis in patients with colorectal cancer undergoing primary tumor resection. Dis Colon Rectum 58:1048–1057. 10.1097/DCR.0000000000000458 26445177 10.1097/DCR.0000000000000458

[11] Shoji F, Takeoka H, Kozuma Y, Toyokawa G, Yamazaki K, Ichiki M et al (2019) Pretreatment prognostic nutritional index as a novel biomarker in non-small cell lung cancer patients treated with immune checkpoint inhibitors. Lung Cancer 136:45–51. 10.1016/j.lungcan.2019.08.006 31437663 10.1016/j.lungcan.2019.08.006

[12] Suzuki E, Kawata N, Shimada A, Sato H, Anazawa R, Suzuki M et al (2023) Prognostic nutritional index (PNI) as a potential prognostic tool for exacerbation of COPD in elderly patients. Int J Chron Obstruct Pulmon Dis 18:1077–1090. 10.2147/COPD.S385374 37309393 10.2147/COPD.S385374 PMC10257926

[13] Zhang J, Xiao X, Wu Y, Yang J, Zou Y, Zhao Y et al (2022) Prognostic nutritional index as a predictor of diabetic nephropathy progression. Nutrients 14:3634. 10.3390/nu14173634 36079889 10.3390/nu14173634 PMC9460356

[14] Wang Y, Jiang Y, Luo Y, Lin X, Song M, Li J et al (2023) Prognostic nutritional index with postoperative complications and 2-year mortality in hip fracture patients: an observational cohort study. Int J Surg 109:3395. 10.1097/JS9.0000000000000614 37526114 10.1097/JS9.0000000000000614 PMC10651254

[15] Zhang J, Yang M, Zhang X, He J, Wen L, Wang X et al (2022) The effectiveness of a co-management care model on older hip fracture patients in China - a multicentre non-randomised controlled study. Lancet Reg Health West Pac 19:100348. 10.1016/j.lanwpc.2021.100348 35141666 10.1016/j.lanwpc.2021.100348 PMC8814766

[16] Katzman R, Zhang M, Ouang Y-Q, Wang Z, Liu WT, Yu E et al (1988) A Chinese version of the Mini-Mental State Examination; impact of illiteracy in a Shanghai dementia survey. J Clin Epidemiol 41:971–8. 10.1016/0895-4356(88)90034-0 3193141 10.1016/0895-4356(88)90034-0

[17] Voeten SC, Nijmeijer WS, Vermeer M, Schipper IB, Hegeman JH (2020) DHFA Taskforce study group. Validation of the Fracture Mobility Score against the Parker Mobility Score in hip fracture patients. Injury 51:395–9. 10.1016/j.injury.2019.10.035 31668574 10.1016/j.injury.2019.10.035

[18] Herdman M, Gudex C, Lloyd A, Janssen M, Kind P, Parkin D et al (2011) Development and preliminary testing of the new five-level version of EQ-5D (EQ-5D-5L). Qual Life Res 20:1727–1736. 10.1007/s11136-011-9903-x 21479777 10.1007/s11136-011-9903-x PMC3220807

[19] Luo N, Liu G, Li M, Guan H, Jin X, Rand-Hendriksen K (2017) Estimating an EQ-5D-5L Value Set for China. Value Health 20:662–669. 10.1016/j.jval.2016.11.016 28408009 10.1016/j.jval.2016.11.016

[20] Cerri AP, Bellelli G, Mazzone A, Pittella F, Landi F, Zambon A et al (2015) Sarcopenia and malnutrition in acutely ill hospitalized elderly: prevalence and outcomes. Clin Nutr 34:745–751. 10.1016/j.clnu.2014.08.015 25263170 10.1016/j.clnu.2014.08.015

[21] Foo MXE, Wong GJY, Lew CCH (2021) A systematic review of the malnutrition prevalence in hospitalized hip fracture patients and its associated outcomes. JPEN J Parenter Enteral Nutr 45:1141–1152. 10.1002/jpen.2211 34169533 10.1002/jpen.2211

[22] Dent E, Hoogendijk EO, Visvanathan R, Wright ORL (2019) Malnutrition screening and assessment in hospitalised older people: a review. J Nutr Health Aging 23:431–441. 10.1007/s12603-019-1176-z31021360 10.1007/s12603-019-1176-z

[23] Hu Y, Yang H, Zhou Y, Liu X, Zou C, Ji S et al (2022) Prediction of all-cause mortality with malnutrition assessed by nutritional screening and assessment tools in patients with heart failure: a systematic review. Nutr Metab Cardiovasc Dis 32:1361–1374. 10.1016/j.numecd.2022.03.009 35346547 10.1016/j.numecd.2022.03.009

[24] Zhang Q, Qian L, Liu T, Ding J-S, Zhang X, Song M-M et al (2021) Prevalence and prognostic value of malnutrition among elderly cancer patients using three scoring systems. Front Nutr 8:738550. 10.3389/fnut.2021.738550 34708064 10.3389/fnut.2021.738550 PMC8544751

[25] Xing H, Xiang D, Li Y, Ji X, Xie G (2020) Preoperative prognostic nutritional index predicts postoperative delirium in elderly patients after hip fracture surgery. Psychogeriatrics 20:487–494. 10.1111/psyg.12511 31951677 10.1111/psyg.12511

[26] Faust LM, Lerchenberger M, Gleich J, Linhart C, Keppler AM, Schmidmaier R et al (2023) Predictive value of prognostic nutritional index for early postoperative mobility in elderly patients with pertrochanteric fracture treated with intramedullary nail osteosynthesis. J Clin Med 12:1792. 10.3390/jcm12051792 36902579 10.3390/jcm12051792 PMC10003114

[27] Dyer SM, Crotty M, Fairhall N, Magaziner J, Beaupre LA, Cameron ID et al (2016) A critical review of the long-term disability outcomes following hip fracture. BMC Geriatr 16:158. 10.1186/s12877-016-0332-0 27590604 10.1186/s12877-016-0332-0 PMC5010762

[28] Campenfeldt P, Ekström W, Al-Ani AN, Weibust E, Greve K, Hedström M (2020) Health related quality of life and mortality 10 years after a femoral neck fracture in patients younger than 70 years. Injury 51:2283–2288. 10.1016/j.injury.2020.06.029 32620326 10.1016/j.injury.2020.06.029

[29] Peeters CMM, Visser E, Van de Ree CLP, Gosens T, Den Oudsten BL, De Vries J (2016) Quality of life after hip fracture in the elderly: a systematic literature review. Injury 47:1369–1382. 10.1016/j.injury.2016.04.018 27178770 10.1016/j.injury.2016.04.018

[30] Munteanu C, Schwartz B (2022) The relationship between nutrition and the immune system. Front Nutr 9:1082500. 10.3389/fnut.2022.1082500 36570149 10.3389/fnut.2022.1082500 PMC9772031

