# Supplementary material for: Prognostic nutritional index (PNI) is an independent predictor for functional outcome after hip fracture in the elderly: a prospective cohort study
Source: Arch Osteoporos. 2024 Nov 5;19(1):107. doi: 10.1007/s11657-024-01469-1 (PMC11538184; doi:10.1007/s11657-024-01469-1)
Supplement: Supplementary file 1 — Supplementary file1 (DOCX 25 KB) [file 11657_2024_1469_MOESM1_ESM.docx]

**Supplementary Table 1. Multiple logistic regression for patients with unrestricted mobility at follow-up time points**

| **Variables** | **OR (95% CI)** | **Estimate** | **S.E** | **Z** | **p-value** |
| --- | --- | --- | --- | --- | --- |
| Discharge |  |  |  |  |  |
| PNI |  |  |  |  |  |
| Lower | Reference |  |  |  |  |
| Higher | 0.45 (0.13-1.43) | -0.80 | 0.61 | -1.32 | 0.188 |
| Age | 1.01 (0.93-1.09) | 0.01 | 0.04 | 0.22 | 0.824 |
| Sex |  |  |  |  |  |
| Female | Reference |  |  |  |  |
| Male | 2.41 (0.65-8.06) | 0.88 | 0.63 | 1.40 | 0.162 |
| BMI | 1.01 (0.86-1.18) | 0.01 | 0.08 | 0.15 | 0.880 |
| Diabetes | 0.85 (0.22-2.77) | -0.16 | 0.63 | -0.25 | 0.801 |
| Hypertension | 2.11 (0.64-8.43) | 0.74 | 0.64 | 1.16 | 0.247 |
| Cognitive impairment | 2.63 (0.37-11.49) | 0.97 | 0.83 | 1.16 | 0.245 |
| Ever or current smoker | 0.26 (0.01-1.62) | -1.34 | 1.11 | -1.20 | 0.229 |
| Fracture type |  |  |  |  |  |
| FNF | Reference |  |  |  |  |
| ITF | 3.62 (1.04-13.58) | 1.29 | 0.64 | 2.00 | 0.045 |
| Anemia | 0.36 (0.09-1.33) | -1.01 | 0.67 | -1.50 | 0.133 |
| LOS | 0.90 (0.66-1.11) | -0.10 | 0.13 | -0.76 | 0.448 |
| **30d** |  |  |  |  |  |
| PNI |  |  |  |  |  |
| Lower | Reference |  |  |  |  |
| Higher | 1.77 (0.88-3.69) | 0.57 | 0.36 | 1.58 | 0.115 |
| Age | 0.98 (0.94-1.03) | -0.02 | 0.02 | -0.80 | 0.424 |
| Sex |  |  |  |  |  |
| Female | Reference |  |  |  |  |
| Male | 2.31 (1.09-4.79) | 0.84 | 0.38 | 2.23 | 0.026 |
| BMI | 0.96 (0.87-1.05) | -0.04 | 0.05 | -0.92 | 0.359 |
| Diabetes | 0.78 (0.35-1.61) | -0.25 | 0.38 | -0.65 | 0.515 |
| Hypertension | 1.06 (0.54-2.09) | 0.05 | 0.34 | 0.16 | 0.875 |
| Cognitive impairment | 0.91 (0.14-3.38) | -0.10 | 0.77 | -0.13 | 0.899 |
| Ever or current smoker | 0.81 (0.29-2.02) | -0.21 | 0.49 | -0.44 | 0.661 |
| Fracture type |  |  |  |  |  |
| FNF | Reference |  |  |  |  |
| ITF | 0.35 (0.14-0.82) | -1.04 | 0.45 | -2.31 | 0.021 |
| Anemia | 0.82 (0.32-1.97) | -0.20 | 0.46 | -0.44 | 0.659 |
| LOS | 0.99 (0.87-1.09) | -0.01 | 0.06 | -0.20 | 0.838 |
| **120d** |  |  |  |  |  |
| PNI |  |  |  |  |  |
| Lower | Reference |  |  |  |  |
| Higher | 1.69 (1.10-2.61) | 0.52 | 0.22 | 2.38 | 0.017 |
| Age | 0.98 (0.95-1.00) | -0.03 | 0.01 | -1.77 | 0.077 |
| Sex |  |  |  |  |  |
| Female | Reference |  |  |  |  |
| Male | 1.96 (1.21-3.19) | 0.67 | 0.25 | 2.73 | 0.006 |
| BMI | 1.00 (0.95-1.06) | 0.00 | 0.03 | 0.09 | 0.925 |
| Diabetes | 1.19 (0.76-1.87) | 0.17 | 0.23 | 0.76 | 0.448 |
| Hypertension | 0.56 (0.37-0.85) | -0.58 | 0.22 | -2.69 | 0.007 |
| Cognitive impairment | 2.07 (0.94-4.46) | 0.73 | 0.39 | 1.84 | 0.065 |
| Ever or current smoker | 1.16 (0.63-2.11) | 0.15 | 0.31 | 0.49 | 0.628 |
| Fracture type |  |  |  |  |  |
| FNF | Reference |  |  |  |  |
| ITF | 0.61 (0.38-0.99) | -0.49 | 0.25 | -1.97 | 0.049 |
| Anemia | 0.92 (0.55-1.55) | -0.08 | 0.26 | -0.32 | 0.751 |
| LOS | 0.95 (0.88-1.02) | -0.05 | 0.04 | -1.29 | 0.197 |
| **1-year** |  |  |  |  |  |
| PNI |  |  |  |  |  |
| Lower | reference |  |  |  |  |
| Higher | 1.07 (0.70-1.62) | 0.30 | 0.22 | 1.34 | 0.179 |
| Age | 0.92 (0.90-0.95) | -0.14 | 0.02 | -8.38 | < 0.001 |
| Sex |  |  |  |  |  |
| Female | Reference |  |  |  |  |
| Male | 1.18 (0.73-1.94) | 0.51 | 0.28 | 1.84 | 0.066 |
| BMI | 0.99 (0.94-1.05) | -0.02 | 0.03 | -0.78 | 0.435 |
| Diabetes | 0.83 (0.53-1.28) | -0.49 | 0.24 | -2.08 | 0.038 |
| Hypertension | 0.69 (0.45-1.06) | -0.47 | 0.23 | -2.05 | 0.040 |
| Cognitive impairment | 0.93 (0.43-2.05) | -0.59 | 0.46 | -1.28 | 0.201 |
| Ever or current smoker | 0.76 (0.42-1.41) | -0.32 | 0.35 | -0.93 | 0.351 |
| Fracture type |  |  |  |  |  |
| FNF | Reference |  |  |  |  |
| ITF | 0.82 (0.51-1.30) | -0.33 | 0.26 | -1.27 | 0.204 |
| Anemia | 0.66 (0.40-1.08) | 0.20 | 0.28 | 0.74 | 0.461 |
| LOS | 0.94 (0.88-1.01) | -0.09 | 0.03 | -2.62 | 0.009 |
| **3-year** |  |  |  |  |  |
| PNI |  |  |  |  |  |
| Lower | Reference |  |  |  |  |
| Higher | 1.35 (0.87-2.09) | 0.30 | 0.22 | 1.33 | 0.179 |
| Age | 0.87 (0.84-0.90) | -0.14 | 0.02 | -8.06 | < 0.001 |
| Sex |  |  |  |  |  |
| Female | Reference |  |  |  |  |
| Male | 1.67 (1.00-2.83) | 0.51 | 0.27 | 1.93 | 0.054 |
| BMI | 0.98 (0.92-1.04) | -0.02 | 0.03 | -0.79 | 0.428 |
| Diabetes | 0.61 (0.38-0.97) | -0.49 | 0.24 | -2.09 | 0.037 |
| Hypertension | 0.62 (0.40-0.97) | -0.47 | 0.23 | -2.10 | 0.036 |
| Cognitive impairment | 0.56 (0.24-1.25) | -0.59 | 0.42 | -1.40 | 0.160 |
| Ever or current smoker | 0.73 (0.38-1.39) | -0.32 | 0.33 | -0.97 | 0.332 |
| Fracture type |  |  |  |  |  |
| FNF | Reference |  |  |  |  |
| ITF | 0.72 (0.44-1.18) | -0.33 | 0.25 | -1.31 | 0.190 |
| Anemia | 1.23 (0.73-2.07) | 0.20 | 0.27 | 0.76 | 0.444 |
| LOS | 0.92 (0.85-0.98) | -0.09 | 0.04 | -2.40 | 0.016 |

Abbreviations: OR, odds ratio; PNI, prognostic nutritional index; S.E, standard error; BMI, body mass index; FNF, femoral neck fracture; ITF, intertrochanteric fracture; LOS, length of stay.
